# Evaluation of Pain-Associated Behavioral Changes in Monoiodoacetate-Induced Osteoarthritic Rats Using Dynamic Weight Bearing Analysis

**DOI:** 10.3390/life14080983

**Published:** 2024-08-06

**Authors:** Devika Kishnan, Erick Orozco Morato, Aydin Calsetta, Kyle M. Baumbauer, Lakshmi S. Nair

**Affiliations:** 1 Department of Biomedical Engineering, University of Connecticut, Storrs, CT 06269, USA; 2 The Cato T. Laurencin Institute for Regenerative Engineering, University of Connecticut, Storrs, CT 06269, USA; 3 Department of Skeletal Biology and Regeneration, University of Connecticut Health Center, Farmington, CT 06030, USA; 4 Department of Physiology and Neurobiology, University of Connecticut, Storrs, CT 06269, USA; 5 Department of Cell Biology and Physiology, University of Kansas Medical Center, Kansas City, KS 66103, USA; 6 Department of Anesthesiology, University of Kansas Medical Center, Kansas City, KS 66103, USA; 7 Department of Orthopedic Surgery, University of Connecticut Health Center, Farmington, CT 06030, USA; 8 Department of Material Science and Engineering, University of Connecticut, Storrs, CT 06269, USA

**Keywords:** monoiodoacetate, dynamic weight bearing, weight percentage, weight ratio, area ratio, acute pain, chronic pain

## Abstract

Pain is the primary clinical indication of osteoarthritis (OA), and behavioral assessments in rodent pain models are widely used to understand pain patterns. These preclinical pain assessments can also help us to understand the effectiveness of emerging therapeutics for prolonged OA pain management. Along with evoked methods like mechanical allodynia and thermal hyperalgesia, non-evoked methods such as dynamic weight bearing (DWB) analysis are valuable tools for behavioral assessments of pain. Both these methods were utilized to study pain-induced behavioral changes in a monoiodoacetate (MIA)-induced osteoarthritic pain model, which is a well-established preclinical OA pain model. However, the utility of DWB analysis as an indicator of long-term pain sensitivity (more than 4 weeks) remains largely unexplored. Understanding the long-term sensitivity of DWB is valuable to study the effectiveness of novel prolonged pain-relieving therapeutics. Here, we studied the dynamic behavioral changes in MIA-induced OA rats over a period of 16 weeks using DWB measurements. Female Sprague Dawley rats were injected in the right knee joint with MIA (3 mg) using X-ray guidance. Multiple dynamic postural evaluations such as ipsilateral weight percentage, paw area, contralateral/ipsilateral weight ratio and area ratio were assessed to understand the behavioral changes. The data showed that the ipsilateral weight bearing percentage alone is not sufficient to assess pain-related behavior beyond 6 weeks. This study shows the advantages and limitations of dynamic weight bearing as an assessment tool for the long-term progression of pain behavior in MIA-induced OA rats.

## 1. Introduction

Osteoarthritis (OA) is a chronic joint disease that causes the degradation of articular cartilage and impaired mobility [1]. OA affects 37.4% of the US population above the age of 60 years [2]. Since joint pain presents as one of the common symptoms of OA, there is a dire need for OA pain management therapies. Current options to manage OA pain include opioids, non-steroidal anti-inflammatory drugs (NSAIDS), hyaluronate injections and corticosteroids; however, their short-term action necessitates multiple/repeated doses to achieve long-term pain relief [3,4,5,6,7]. Thus, there is a significant need for long-acting pain-relieving analgesics to effectively manage OA pain. In addition, pre-clinical models and tools that can evaluate and measure long-term OA pain are critical to the development of these novel prolonged therapies. Among the preclinical rodent OA models, monoiodoacetate (MIA) is the most commonly used model for studying OA pain, largely because of the relatively quick onset of OA pain compared to other models such as mechanical destabilization, tissue transection or enzymatically induced OA models [8,9,10]. MIA-induced OA produces dose-dependent cartilage degradation and results in rapid and progressive disease progression upon induction [11,12,13]. 

Both evoked and non-evoked pain assessment methods are currently used to assess pain in preclinical models. Evoked methods for assessing mechanical allodynia, such as Von Frey filaments, use an external stimulus to elicit a response from the subject, while non-evoked tools attempt to capture spontaneous pain behaviors [14]. Combining evoked tools to assess changes to sensitivity with non-evoked pain analysis and whole body dynamics could present a comprehensive understanding of the pain experience in preclinical models [15]. One of the methods to assess non-evoked spontaneous pain is the rat grimace scale (RGS), which captures known rat facial expressions using photographs [16,17]. Similarly, automated non-evoked systems such as CatWalk^TM^ XT and Digigait^TM^ detect gait kinetics and estimate weight bearing information from photographs [16,18,19,20,21]. Using CatWalk analysis, Han et al. studied parameters such as stand time, paw print area and swing speed and reported that all these parameters were significantly increased in the MIA (3 mg)-induced OA rodent model compared to saline groups for 6 weeks. The open field locomotion test is another tool to measure general locomotor activity and anxiety-driven behaviors in rodents; however, it does not provide weight bearing information [22,23,24]. Weight bearing can provide a quantitative assessment of sensitivity towards OA pain, as animals tend to be protective of ipsilateral limbs or paws. Static weight bearing or an incapacitance test, in which rodents are placed in a restraining device with their hind limbs placed on two pressure sensors to measure weight distribution, are commonly used in this aspect. In spite of providing quantifiable data, the placement of rodents in a restricted environment to perform this study may introduce confounding factors due to the increased stress that animals are subjected to and the innate explorative nature of the species [25,26].

**Figure 1:**
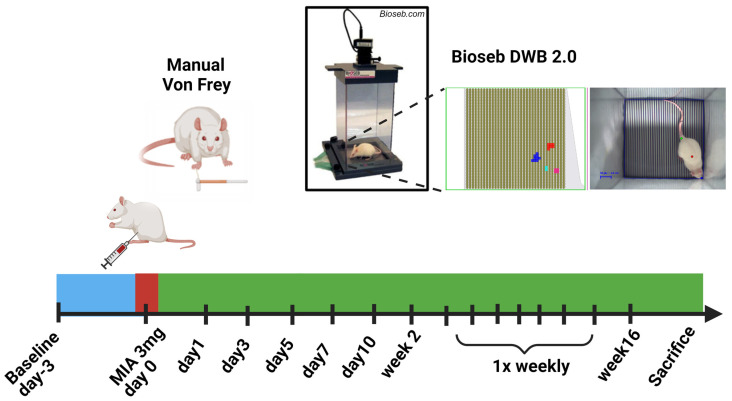
Experimental Timeline. Baseline pre-injury measurements were conducted 3–5 days before OA induction. At day 0, female Sprague Dawley rats were injected in the right knee joint with monoiodoacetate using X-ray guidance. Manual Von Frey and dynamic weight bearing (DWB) analyses were conducted on day 1, day 3, day 5, day 7, day 10 and day 14 and weekly thereafter until 16 weeks. Created with Biorender.com (accessed on 4 August 2024).

Dynamic Weight Bearing (DWB) is a user-independent pressure-sensor-based method for measuring unrestrained weight bearing information from rodents [27]. Here, the rodents are acclimated to an enclosed chamber (Figure 1) and allowed to freely explore the chamber, enhancing the potential for measuring unrestricted weight-bearing behavior [16,28]. This allows the detection of weight exerted by all four paws of the rodents, as well as providing a quantitative estimate of other variables such as the time of contact of the paw with the floor and the paw area on the floor. In addition to weight bearing, a wide range of temporal and spatial measurements can be used to understand the pain behavior. Few recent studies have investigated the changes in weight bearing in MIA-induced OA rats for a short period of 2–4 weeks to understand short-term behavioral changes [29,30,31,32]. The goal of this study was to understand longer term (up to 16 weeks) non-evoked pain behavior in MIA (3 mg)-induced OA rats using multiple dynamic postural evaluations such as weight bearing, paw area, weight and area ratios. 

## 2. Materials and Methods

### 2.1. Animals

Female Sprague Dawley rats weighing between 200 and 250 g were used in the study. The Sprague Dawley rats, 8 weeks old, were ordered from Charles River Laboratories, USA. Animals were housed in pairs in a 12 h light/dark cycle and were provided with food and water ad libitum. The animal study protocols were approved by the Institutional Animal Care and Use Committee (IACUC) at the University of Connecticut Health. The animals were acclimated to the room in which all behavioral assessments were performed. The authors complied with ARRIVE guidelines.

### 2.2. Induction of Osteoarthritis

In order to induce OA, rats were injected in the right knee joint with sodium salt of monoiodoacetate (Sodium iodoacetate, Sigma-Aldrich, St. Louis, MO, USA) under X-ray (OEC Mini6600 C-Arm machine, GE, Boston, MA, USA) guidance. To optimize the injection volume to contain MIA within the synovial cavity, different volumes were tested with an optical contrast agent OptiRay 240 (Guerbet, Raleigh, NC, USA), and 30 μL was determined as the optimal volume. Animals were given an X-ray-guided injection of 3 mg MIA in 30 μL of 0.9% saline (Sigma-Aldrich, St. Louis, MO, USA) using a 29-gauge needle. A total of 6 animals were used for the study. The sample size was determined with power analysis using paw weight ratio data from Kobayashi et al. [11]. 

### 2.3. In Vivo Evoked Pain Assessment

Mechanical Allodynia was measured using manual Von Frey filaments. The animals were acclimated for 10 min on the Von Frey grid apparatus before each measurement. Von Frey filaments from #2 to #10 were used to assess allodynia. An ascending up and descending down method was used to estimate the lowest force that elicited a paw withdrawal response. The Von Frey filaments were applied to the rats’ paws in order of increasing strength until a paw withdrawal was observed. After this, the next filament, with increased strength, was applied to eliminate a false positive. Following this, the filaments were applied in the order of decreasing strength to measure the smallest force that led to a response. The Von Frey measurements were carried out at baseline pre-MIA injection and post-MIA injection on day 1, day 3, day 5, day 7, day 10 and day 14 and weekly until 16 weeks. Behavioral assessments at each time point were taken in the same room for the entire period of the study.

### 2.4. Dynamic Weight Bearing Analysis

Dynamic Weight Bearing 2.0 apparatus (Bioseb, Vitrolles, France) was used. The DWB 2.0 equipment consists of a plexiglass chamber (Figure 1) with a pressure sensor floor to detect the force exerted by the rodent’s paws. A camera at the top of the chamber captures the movements of the rodent to assist in the correct assignment of pixels to the corresponding paws. In accordance with the manufacturer’s guidelines, the animals were acclimated to the equipment for three minutes, followed by five minutes of weight bearing behavioral data acquisition. The software carries out auto-scoring to assign paw names to the detected pixels and allows for manual scoring by the analysts. The manual scoring was performed by going through each frame of the acquisition and verifying that the scoring had assigned the paws correctly. After the manual scoring has been completed, the software produces thirty-six variables which can be used to assess the gait behavior of the rodent. The thirty-six variables derive from three broad categories pertaining to (a) time, (b) weight and (c) area. Time measurements are associated with the time of contact of each paw and the sensor. The time measurement is valuable as increased pain sensitivity in the feet or joints can lead to animals not placing the paw on the sensor floor for a prolonged time. Weight bearing is the most straightforward measurement and detects the force exerted by each paw or combination of paws on the sensor floor. Since the sensor floor comprises of multiple mini sensor units, the software can determine the effective paw area by detecting the number of sensors activated when a paw is placed on the sensor floor. Thus, the area measurement helps to determine quantifiable changes in the paw area, thereby capturing behavioral traits as the rodents often protect their paws with increased pain sensitivity. The weight bearing assessments were performed pre-MIA injection (referred to as baseline) and post-MIA injection on day 1, day 3, day 5, day 7, day 10 and day 14 and weekly there onwards until week 16 (Figure 1). Behavioral assessments at each time point were taken in the same room for the entire period of the study. In this study, baseline (pre-injury) levels were used as the control. The ipsilateral paw was also compared to the contralateral paw of the same animal at each time point to account for age-matched control.

#### Parameters Studied

Mobile vs. Immobile: If the animal’s posture is undisturbed for greater than 1000 milliseconds (an adjustable threshold), it is classified as immobile; otherwise, it is classified as mobile or moving.

Weight Bearing: Weight bearing on each paw is calculated as the weight percentage, as below.

$$Weight\;Percentage\left(\%\right)=\frac{Force\;exerted\;by\;each\;paw}{Total\;weight\;of\;animal}\times 100$$

Weight Ratio: Weight Ratio is calculated as below.

$$Weight\;Ratio=\frac{Weight\;\%\;by\;Left\;paw\;(Contralateral)}{Weight\;\%\;by\;Right\;Paw\;(Ispilateral)}$$

### 2.5. Histology of the Knee Joints

Animals were sacrificed at the end of the study (16 weeks). Left and right knees were collected and kept in 10% Formalin (Fisherbrand, ThermoFisher, Waltham, MA, USA) in a cold storage room for 5–7 days. The knees were washed in phosphate-buffered saline (BioRad, Hercules, CA) for 1 h and this process was repeated three times. The washed knees were decalcified in Cal-Ex (ThermoFisher, Waltham, MA, USA) for three days at 4 °C, and the Cal-Ex solution was changed every day. Post decalcification, the knees were stored in 100% ethanol (Fisherbrand, Thermofisher, Waltham, MA, USA) at 4 °C, until processing. For processing, the knees were embedded in paraffin and sectioned. The sections were stained with Haemotoxylin and Eosin (H&E) stain and Safranin O. 

### 2.6. Statistical Analysis

Comparisons of all timepoints to baseline (prior to injury) measurements were carried out using repeated measures one-way ANOVA and Dunnett’s correction for multiple comparisons. Comparisons between variables across various time points were carried out with two-way ANOVA and a post hoc paired Student’s *t*-test with a False Discovery Rate (FDR) approach for multiple comparisons. The data were plotted as mean ± standard deviation(SD). Statistical analysis was conducted with N = 6. The mean, standard deviationfor all data in the plots and the numerical *p*-values for all data are provided in the Supplementary document. Statistical analysis was carried out using software GraphPad Prism 10.1.2.

## 3. Results

### 3.1. Mechanical Allodynia in MIA-Induced OA Rats

Figure 1 shows the experimental timeline for evaluating pain-related behaviors in a monoiodoacetate model using manual Von Frey measurements and dynamic weight bearing apparatus. The baseline measurements were conducted 3–5 days before the MIA injection. Behavioral assessments were carried out on day 1, day 3, day 5, day 7, day 10 and day 14 and weekly for 16 weeks.

**Figure 2:**
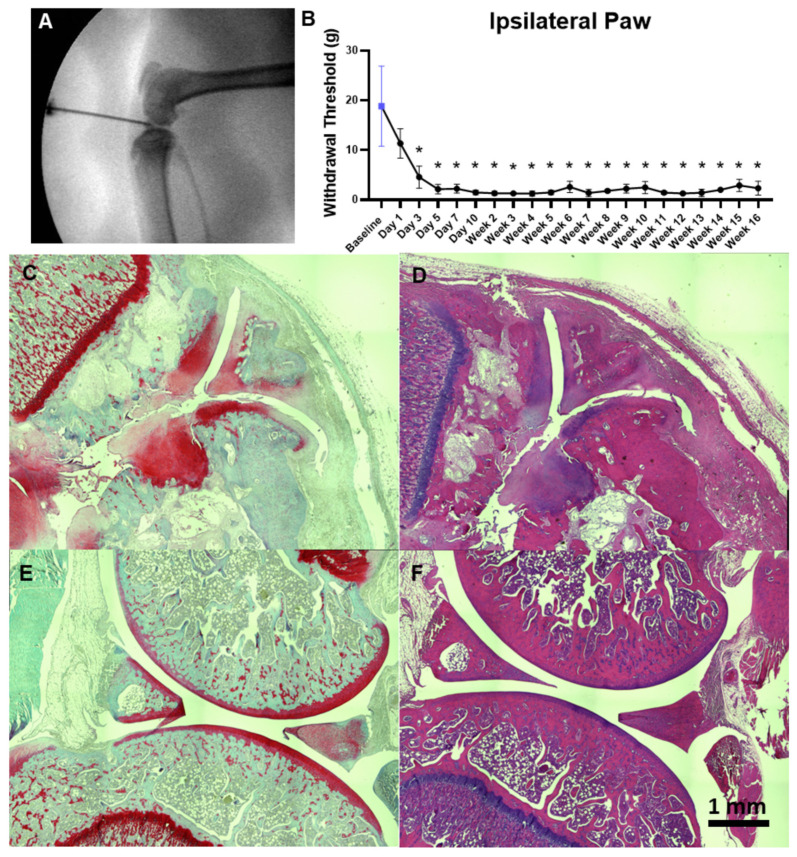
Persistent mechanical allodynia and unilateral knee joint degeneration in the MIA model. (**A**). An X-ray image of a 29-gauge insulin syringe injected into the right knee joint of a rat. X-ray guidance allows visualization of the injection location and enhances the precision of injections. (**B**). Manual Von Frey measurements in MIA-injected rats show significant pain sensitivity from day 5 onwards and persistent mechanical allodynia for 16 weeks. The blue symbol corresponds to baseline measurements. Significance denoted by ‘*’ *p*< 0.05, with repeated measures one-way ANOVA. (**C**). Safranin-O-stained section of ipsilateral knee joint at 16 weeks. (**D**). H&E-stained knee section for ipsilateral knee joint at 16 weeks. Stained sections show that the ipsilateral joint is severely degenerated with cartilage degeneration and growth plate disruption (**C**) and infiltration of inflammatory cells (**D**). (**E**). Safranin O-stained section of contralateral joint at 16 weeks. (**F**). H&E-stained section of contralateral knee at 16 weeks. The contralateral joints show a preserved cartilage layer, indicating that MIA-induced osteoarthritis led to a unilateral knee OA.

Figure 2A shows the X-ray-guided intra-articular injection of 30 μL of 3 mg MIA. As can be seen, X-ray guidance allows the visualization of injection location and enhances the precision of intra-articular injections. Figure 2B shows the mechanical allodynia of the ipsilateral paw, as measured using manual Von Frey filaments. Statistically significant decreases in the paw withdrawal threshold were observed from day 5 (2.133 ± 0.961 (s.d)) onwards compared to the baseline (18.833 ± 8.06 (s.d)) and persisted until the end of the study (16 weeks post injection), demonstrating the establishment of consistent longer-term mechanical allodynia. Mean and standard deviation at each time point are provided in Supplementary Table S1.

### 3.2. Histology of MIA-Induced OA Knee Joints at 16 Weeks Post Injection

Figure 2C–F show the Safranin O- and H&E-stained sections of ipsilateral and contralateral knees at 16 weeks post injection. The stained sections displayed cartilage degeneration, sub chondral bone remodeling (Safranin O) and the infiltration of inflammatory cells (H&E) in the ipsilateral joint by 16 weeks (Figure 2C,D). The contralateral joint displayed no cartilage degeneration, demonstrating the presence of unilateral OA at 16 weeks post induction (Figure 2E,F). 

### 3.3. Weight Bearing Assessment of MIA-Induced OA Rats

**Figure 3:**
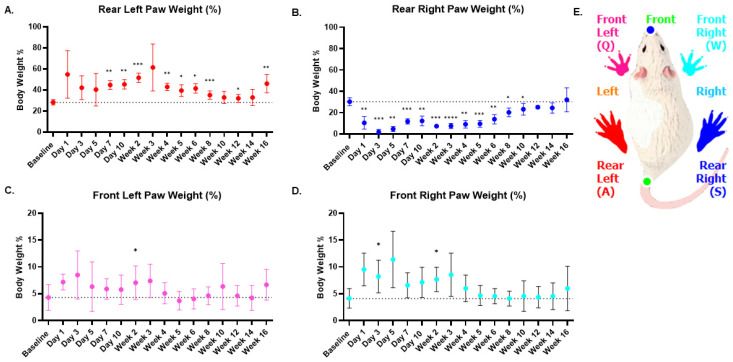
Weight bearing percentages of all four rodent paws. (**A**). Weight bearing by the rear left paw (contralateral) for 16 weeks. (**B**). Weight bearing by the rear right (ipsilateral) paw for 16 weeks. (**C**). Weight bearing by the front left paw for 16 weeks. (**D**). Weight bearing by the front right paw for 16 weeks. (**E**). The illustration indicates the color legend coding for each of the paws. The rear right (ipsilateral) weight percentage shows significant reduced weight bearing compared to pre-injury levels for 10 weeks. The contralateral weight percentage shows increased weight bearing and displays significant differences compared to baseline for 16 weeks using repeated measures one-way ANOVA and Dunnett’s multiple comparison test. Significance denoted by ‘*’ *p* < 0.05, ‘**’ *p* < 0.01 and ‘***’ *p* < 0.001 and ‘****’ *p* < 0.0001.

Weight bearing information collected at different timepoints can help to quantitatively understand the gait of animals. Figure 3 elucidates the weight bearing measurements of each paw of the animal. The rodent image provides the colored legend corresponding to each paw (Figure 3E). The ipsilateral paw (rear right) showed a statistically significant reduction in weight bearing for a period of 10 weeks compared to the baseline (Figure 3B). For example, the ipsilateral paw weight percentage at day 3 was 2.163 ± 2.228 (s.d) compared to the baseline weight percentage of 30.382 ± 3.7 (s.d). This is also reflected in the contralateral paw (rear left), as it showed compensatory enhanced weight bearing from baseline until 16 weeks (Figure 3A). Moreover, as shown in the figures, both these measurements displayed temporal changes over 16 weeks. Interestingly, the ipsilateral paw weight bearing showed an upward trend starting from 6 weeks (13.930 ± 4.319 (s.d)), indicating that the animals slowly began to adjust their gait from the 6-week time point onwards. The front left (Figure 3C) and front right (Figure 3D) weight percentages displayed statistically significant differences compared to baseline only in the first 2 weeks post injury and were mostly consistent in their weight percentages after 2 weeks. 

By assessing the weight bearing on all four paws of the rodent (Figure 3A–D), the study demonstrated that the weight bearing of the contralateral (rear left) paw mirrors the ipsilateral (rear right) paw and shows decreased weight bearing after 6 weeks, indicating less compensation by the contralateral paw as the animal modulates its gait pattern. Mean and standard deviation at each time point are provided in Supplementary Tables S2–S5.

### 3.4. Comparison of Ipsilateral–Contralateral Weight Bearing

**Figure 4:**
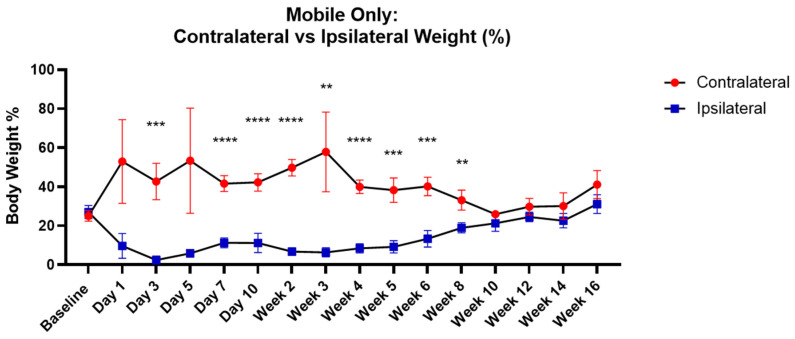
Comparison between contralateral and ipsilateral paw weight percentages at specific time points. The figure compares the contralateral and ipsilateral weight percentages at each specific time point, with the mobility threshold applied.There were significant differences in weight bearing between the ipsilateral (rear right) and contralateral paws (rear left) from day 3 to 8 weeks. Significance denoted by ‘**’ *p* < 0.01, ‘***’ *p* <0.001 and ‘****’ *p* <0.0001 with two-way ANOVA with post hoc paired Student’s *t*-test with False Discovery Rate (FDR) approach for multiple comparisons.

In addition to assessing the weight bearing of each paw over the 16 weeks compared to the pre-injury baseline levels (Figure 3), this study also investigated the measurement of weight bearing at each specific time point between the ipsilateral and contralateral paws, respectively, with the mobility threshold applied (Figure 4). As can be seen, there were statistically significant differences between weight bearing percentages for 8 weeks. For example, on day 7, the contralateral weight percentage (41.652 ± 4.054 (s.d)) was significantly higher than the ipsilateral weight percentage (11.312 ± 2.513 (s.d)), whereas at 10 weeks, the contralateral (25.928 ± 1.741 (s.d)) and ipsilateral (21.317 ± 4.079 (s.d)) weight percentages showed no significant differences. Similarly, the comparisons between the ipsilateral and age-matched contralateral weight percentages showed significant differences up to 8 weeks, after which there was no significant difference between the forces exerted by the paws. Mean and standard deviation at each time point are provided in Supplementary Table S6. 

### 3.5. Evaluation of Paw Area

**Figure 5:**
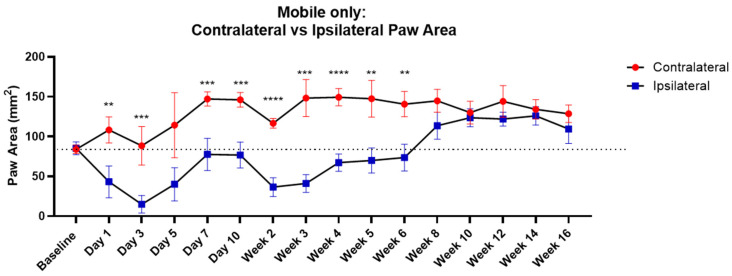
Comparison of contralateral and ipsilateral paw area at specific time points. The figure displays the contralateral paw area and the ipsilateral paw area at each specific time point, with the mobility threshold applied. The ipsilateral (rear right) paw area showed significantly reduced paw area at time points day 1, day 3 and day 7, which corresponds to a behavior pattern of rodents protecting their injured paws during this acute time period post injury. The comparison of ipsilateral (rear right) and contralateral (rear left) paw area showed significant differences for 6 weeks despite animal growth over time. Significance denoted by ‘**’ *p* < 0.01, ‘***’ *p* < 0.001 and ‘****’ *p* < 0.0001 with two-way ANOVA with post hoc paired Student’s *t*-test with False Discovery Rate (FDR) approach for multiple comparisons.

This study also investigated the pixels activated by the contact of each of the animal paws so as to detect (a) the time of contact of the paw with the sensors and (b) the effective area of the paw placed on the sensors. The paw area can be a useful measurement as the OA rats often tend to curl up the paws as an indicator of pain, and hence with greater paw sensitivity, the paw area would be reduced. Paw area analysis at the time points of day 1, day 3 and day 7 (Figure 5; blue squares) shows that animals had reduced paw area on the ipsilateral right paw compared to the contralateral paw (Figure 5, red circles). For example, animals showed significantly reduced ipsilateral paw area on day 3 (14.923 ± 10.974 (s.d)) compared to contralateral paw area (88.385 ± 24.9 (s.d)) on the same day. The paw area also showed temporal changes up until 6 weeks. The mean and standard deviation at each time point are provided in Supplementary Table S7.

### 3.6. Evaluation of Weight and Area Ratio

**Figure 6:**
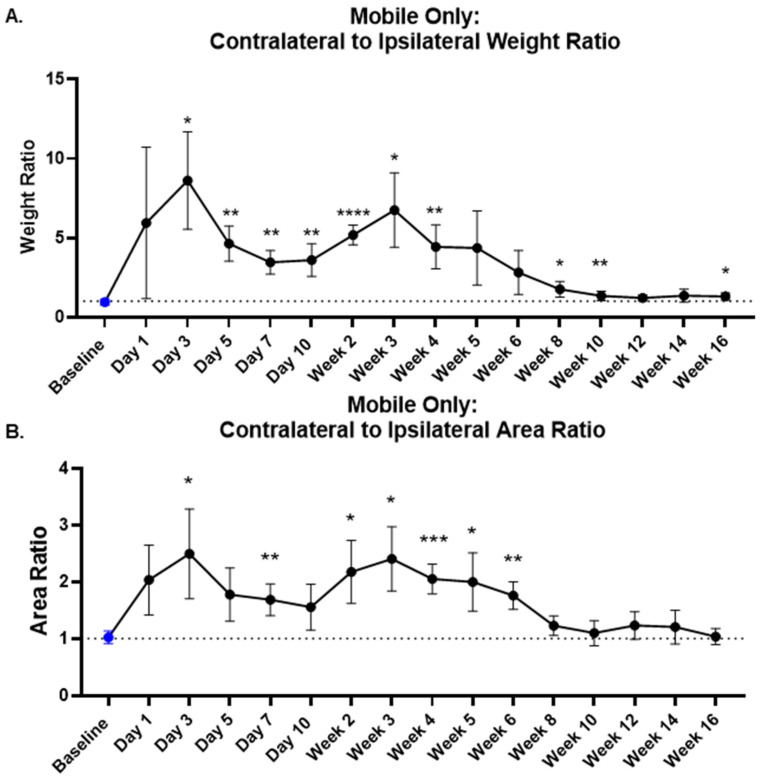
Weight Ratio and Area Ratio. (**A**) The plot displays the contralateral/ipsilateral weight ratio from the rodents’ weight bearing analysis with the mobility threshold applied. The blue symbol corresponds to the baseline measurements. The weight ratio was able to show significant difference from baseline behavior for 16 weeks. The weight ratio shows bimodal temporal changes, with the first peak at day 3 and the second peak at day 21. (**B**). The plot displays area ratio with contralateral and ipsilateral paw area. The area ratio also shows bimodal changes, with peaks at day 3 and day 21. Significance denoted by ‘*’ *p* < 0.05, ‘**’ *p* < 0.01, ‘***’ *p* < 0.001 and ‘****’ *p* < 0.0001 with repeated measures one-way ANOVA and Dunnett’s multiple comparison test.

This study also investigated the utility of the weight and area ratio to access long-term pain when the animals are mobile. Figure 6A shows the rear contralateral/rear ipsilateral weight ratios across the period of the study, and statistical differences up to 16 weeks were found when compared to the baseline. The study showed temporal bimodal changes in the weight ratio, with the first peak occurring at day 3 (8.607 ± 3.07 (s.d)) compared to baseline (0.942 ± 0.186 (s.d)), followed by another peak at day 21 (6.750 ± 2.343 (s.d)). An increase in the weight ratio is indicative of increased weight bearing on the contralateral, and hence can be interpreted as animals experiencing more pain. The rear contralateral/rear ipsilateral area ratio (Figure 6B) showed a similar temporal change, displaying a peak pain level at day 3 (2.495 ± 0.79 (s.d)) compared to baseline (1.023 ± 0.113 (s.d)), followed by a decrease in pain levels until day 10 (1.555 ± 0.403 (s.d)). This area ratio also indicates a second phase of rising pain levels, starting at day 10 and peaking at day 21 (2.405 ± 0.568 (s.d)). Mean and standard deviation at each time point are provided in Supplementary Tables S8 and S9.

## 4. Discussion

This study demonstrated the advantages of using multiple dynamic postural evaluations to understand the longer-term (16 week) behavioral changes in MIA (3 mg)-induced OA rats. The most commonly reported dynamic weight bearing variable in the MIA-induced OA model was the ipsilateral paw weight. Using dynamic weight bearing measurements, Rashid et al. showed significant differences in ipsilateral paw weight in MIA-induced OA rats in a 4-week study [29]. In this 16-week study, significantly reduced weight bearing of the ipsilateral paw compared to the baseline levels was observed for up to 10 weeks. The use of a mobility threshold eliminates the data from when the animals were completely immobile, such as when they were sitting. The application of the mobility threshold further increased the sensitivity and allowed us to understand changes in weight bearing until 16 weeks. Another finding of this study was the longer-term gait adjustment in the animals, resulting in increased weight bearing on the ipsilateral paw after the 6-week time point. This was corroborated by the compensatory weight bearing observed in the contralateral paw. The increased weight bearing of the ipsilateral paw from 6 weeks onwards was rather unexpected, as histological analysis confirmed significant damage of the ipsilateral knee with complete destruction of the cartilage tissue and subchondral bone remodeling at 16 weeks. The study therefore shows the limitation of using the ipsilateral weight percentage as the lone measurement for longer-term pain analysis.

The study also revealed ipsilateral paw area as an effective measurement, as OA pain can prevent the complete placement of the paw on the sensor floor area. However, since the parameter showed significant differences for only up to 6 weeks, the utility of this for longer-term pain analysis is limited. The time of contact of the paw is another potential measurement; however, no significant differences were observed in this study. 

As can be seen, the paw weight and area measurements show dynamic temporal changes. To investigate further, the weight ratio of contralateral to ipsilateral paws was analyzed. Previous studies have reported that MIA-induced OA models show acute pain within the first 7 days and chronic pain afterwards [24,33,34,35,36]. We observed a temporal increase in pain levels, peaking at day 3 during the acute period of MIA, followed by a decrease in pain levels until day 10. After day 10, there was a second wave of increased pain level, peaking by day 21, possibly indicative of the chronic phase of the pain behavior. An analysis of the area ratio between contralateral and ipsilateral paws showed similar temporal changes, indicating a first peak by day 3 and a secondary peak by day 21. Dynamic weight bearing can serve as a valuable non-evoked method when utilized with other pain assessment tools to assess longer-term pain behavior in rodents. The use of variables such as paw weight, paw area and weight ratios and the use of mobile/immobile thresholds, along with weight bearing, could increase the data analytics. The study demonstrated that dynamic postural evaluations can be used to detect pain-related behaviors in a MIA (3 mg)-induced pain model for 10 weeks. 

## 5. Conclusions

This study showed that the use of a single variable such as weight bearing may not provide a comprehensive understanding of the longer-term gait assessment in a MIA-induced OA rat model. The use of multiple dynamic postural evaluations could make dynamic weight bearing a valuable complementary tool for assessing pain-related behaviors in a MIA-induced rodent model.

## Data Availability

The data presented in this study are available in the Supplementary Materials.

## Supplementary Materials

The following supporting information can be downloaded at: https://www.mdpi.com/article/10.3390/life14080983/s1, Table S1: Mechanical Allodynia with Von Frey measurements. Statistical analysis with one-way ANOVA with Geisser-Greenhouse correction and Dunnet’s correction for multiple comparisons; Table S2: Rear Left (Contralateral) Paw Weight Percentage. Statistical analysis with Repeated Measures one-way ANOVA with Geisser-Greenhouse correction and Dunnet’s correction for multiple comparisons; Table S3: Rear Right (Ipsilateral) Paw Weight Percentage. Statistical analysis with Repeated Measures one-way ANOVA with Geisser-Greenhouse correction and Dunnet’s correction for multiple comparisons; Table S4: Front Left Paw Weight Percentage. Statistical analysis with Repeated Measures one-way ANOVA with Geisser-Greenhouse correction and Dunnet’s correction for multiple comparisons; Table S5: Front Right Paw Weight Percentage. Statistical analysis with Repeated Measures one-way ANOVA with Geisser-Greenhouse correction and Dunnet’s correction for multiple comparisons; Table S6: Contralateral and Ipsilateral weight percentage: Mobile only. Statistical analysis with 2-way ANOVA and post-hoc paired *t*-test with FDR approach for multiple comparisons; Table S7: Contralateral and Ipsilateral paw area: Mobile only. Statistical analysis with 2-way ANOVA and post-hoc paired *t*-test with FDR approach for multiple comparisons; Table S8: Contralateral to Ipsilateral Weight Ratio: Mobile Only. Statistical analysis with one-way ANOVA with Geisser-Greenhouse correction and Dunnet’s correction for multiple comparisons; Table S9: Contralateral to Ipsilateral Area Ratio: Mobile Only. Statistical analysis with one-way ANOVA with Geisser-Greenhouse correction and Dunnet’s correction for multiple comparisons.
