# Supplementary material for: Evaluation of Pain-Associated Behavioral Changes in Monoiodoacetate-Induced Osteoarthritic Rats Using Dynamic Weight Bearing Analysis
Source: Life (Basel). 2024 Aug 6;14(8):983. doi: 10.3390/life14080983 (PMC11355379; doi:10.3390/life14080983)
Supplement: Supplementary file 1 [file life-14-00983-s001.zip › life-3133105-supplementary.pdf]

LONG TERM PAIN ASSOCIATED BEHAVIORAL CHANGES IN OSTEOARTHRITIC RAT MODEL USING DYNAMIC WEIGHT BEARING ANALYSIS

Supplementary Data:

S1. Mechanical Allodynia with Von Frey measurements. Statistical analysis with one-way ANOVA with Geisser-Greenhouse correction and Dunnet's correction for multiple comparisons.

ANOVA table	F (DFn, DFd)	P value
Across time (between columns)	F (1.605, 8.027) = 25.72	P=0.0005

Mean, Standard Deviation and p-value at each timepoint are given below:

Timepoint	Mean	SD	Comparison with Baseline: p-value
Day 1	11.333	2.944	0.2905
Day 3	4.567	2.229	0.0689
Day 5	2.133	0.961	0.0212
Day 7	2.233	0.898	0.0391
Day 10	1.467	0.450	0.0255
Day 14	1.300	0.395	0.0267
Week 3	1.267	0.207	0.0233
Week 4	1.233	0.480	0.0224
Week 5	1.467	0.450	0.0219
Week 6	2.567	1.134	0.0209
Week 7	1.367	0.709	0.0212
Week 8	1.800	0.310	0.0274
Week 9	2.233	0.898	0.0234
Week 10	2.467	1.218	0.0219
Week 11	1.433	0.320	0.025
Week 12	1.267	0.207	0.0233
Week 13	1.400	0.738	0.0204
Week 14	2.000	0.000	0.028
Week 15	2.900	1.225	0.0484
Week 16	2.333	1.389	0.0193

S2. Rear Left (Contralateral) Paw Weight Percentage. Statistical analysis with Repeated Measures one-way ANOVA with Geisser-Greenhouse correction and Dunnet's correction for multiple comparisons.

ANOVA table	F (DFn, DFd)	P value
Across time (between columns)	F (2.841, 14.02) = 4.559	0.021

Mean, Standard Deviation and p-value at each timepoint are given below:

Timepoint	Mean	SD	Comparison with Baseline: p-value
Day 1	28.275	2.559	0.1989
Day 3	54.810	22.636	0.2242
Day 5	42.142	11.305	0.612
Day 7	40.346	15.459	0.0063
Day 10	44.772	4.135	0.0032
Day 14	45.415	4.445	0.0007
Week 3	51.612	4.545	0.1048
Week 4	61.463	22.333	0.0035
Week 5	42.953	3.480	0.0146
Week 6	39.435	5.531	0.0109
Week 8	41.498	4.439	0.0009
Week 10	35.128	4.015	0.2695
Week 12	32.900	5.779	0.0404
Week 14	32.010	3.765	0.6008
Week 16	32.910	7.649	0.0091

S3. Rear Right (Ipsilateral) Paw Weight Percentage. Statistical analysis with Repeated Measures one-way ANOVA with Geisser-Greenhouse correction and Dunnet’s correction for multiple comparisons.

ANOVA table	F (DFn, DFd)	P value
Across time (between columns)	F (2.643, 13.04) = 28.90	<0.0001

Mean, Standard Deviation and p-value at each timepoint are given below:

Timepoint	Mean	SD	Comparison with Baseline: p-value
Day 1	10.557	5.816	0.0044
Day 3	2.163	2.228	0.0001
Day 5	4.800	2.350	0.0032
Day 7	11.857	2.521	0.0007
Day 10	12.375	4.467	0.0023
Day 14	7.440	1.487	<0.0001
Week 3	7.608	2.379	<0.0001
Week 4	9.237	3.546	0.0012
Week 5	9.672	3.189	0.0007
Week 6	13.930	4.319	0.0036
Week 8	20.350	4.040	0.0407
Week 10	23.268	5.484	0.04
Week 12	25.183	1.323	0.1585

Week 14	24.502	4.863	0.0921
Week 16	32.147	11.213	>0.9999

S4. Front Left Paw Weight Percentage. Statistical analysis with Repeated Measures one-way ANOVA with Geisser-Greenhouse correction and Dunnet's correction for multiple comparisons.

ANOVA table	F (DFn, DFd)	P value
Across time (between columns)	F (3.228, 15.93) = 2.778	0.072

Mean, Standard Deviation and p-value at each timepoint are given below:

Timepoint	Mean	SD	Comparison with Baseline: p-value
Day 1	7.173	1.459	0.4766
Day 3	8.495	4.493	0.2227
Day 5	6.332	4.627	0.8582
Day 7	5.897	1.899	0.421
Day 10	5.768	2.726	0.4288
Day 14	7.050	3.105	0.0416
Week 3	7.387	3.108	0.2164
Week 4	5.077	1.958	0.992
Week 5	3.690	1.736	0.9956
Week 6	4.043	1.869	0.9982
Week 8	4.635	1.647	>0.9999
Week 10	6.362	4.300	0.8558
Week 12	4.607	1.893	0.9995
Week 14	4.220	2.303	>0.9999
Week 16	6.672	2.862	0.6824

S5. Front Right Paw Weight Percentage. Statistical analysis with Repeated Measures one-way ANOVA with Geisser-Greenhouse correction and Dunnet's correction for multiple comparisons.

ANOVA table	F (DFn, DFd)	P value
Across time (between columns)	F (3.318, 16.37) = 5.138	0.0094

Mean, Standard Deviation and p-value at each timepoint are given below:

Timepoint	Mean	SD	Comparison with Baseline: p-value
Day 1	9.525	3.039	0.1651
Day 3	8.225	3.054	0.013
Day 5	11.398	5.258	0.1382
Day 7	6.587	2.320	0.4078
Day 10	7.135	2.814	0.123
Day 14	7.690	2.258	0.0478

Week 3	8.540	4.035	0.1165
Week 4	6.012	2.476	0.6451
Week 5	4.670	1.865	0.8572
Week 6	4.552	1.418	0.9987
Week 8	4.127	1.387	>0.9999
Week 10	4.553	2.833	>0.9999
Week 12	4.368	1.990	>0.9999
Week 14	4.545	2.491	0.9997
Week 16	5.993	4.134	0.562

S6. Contralateral and Ipsilateral weight percentage: Mobile only. Statistical analysis with 2-way ANOVA and post-hoc paired t-test with FDR approach for multiple comparisons.

ANOVA table	F (DFn, DFd)	P value
Time x Column Factor	F (15, 150) = 13.77	P<0.0001
Time	F (3.191, 31.91) = 2.341	P=0.0885
Column Factor	F (1, 10) = 257.8	P<0.0001

Mean, Standard Deviation and p-value at each timepoint are given below:

Timepoint	Contralateral Mean	Contralateral SD	Ipsilateral Mean	Ipsilateral SD	p-value
Baseline	25.065	2.723	27.043	3.302	ns
Day 1	52.982	21.431	9.672	6.338	ns
Day 3	42.698	9.345	2.463	2.013	0.000107
Day 5	53.352	26.920	5.897	1.952	ns
Day 7	41.652	4.054	11.312	2.513	0.000046
Day 10	42.257	4.467	11.220	4.902	0.000049
Day 14	49.773	4.196	6.783	1.887	<0.000001
Week 3	57.893	20.398	6.378	2.377	0.001666
Week 4	39.967	3.452	8.467	2.347	0.000024
Week 5	38.275	6.274	9.243	3.135	0.000383
Week 6	40.183	4.688	13.385	4.231	0.000126
Week 8	33.138	5.133	18.955	2.562	0.004714
Week 10	25.928	1.741	21.317	4.079	ns
Week 12	29.820	4.256	24.530	2.403	ns
Week 14	30.120	6.780	22.652	3.713	ns
Week 16	41.097	7.165	31.145	4.744	ns

S7. Contralateral and Ipsilateral paw area: Mobile only. Statistical analysis with 2-way ANOVA and post-hoc paired t-test with FDR approach for multiple comparisons.

ANOVA table	F (DFn, DFd)	P value
Time x Column Factor	F (15, 150) = 13.77	P<0.0001
Time	F (3.191, 31.91) = 2.341	P=0.0885
Column Factor	F (1, 10) = 257.8	P<0.0001

Mean, Standard Deviation and p-value at each timepoint are given below:

Timepoint	Contralateral Mean	Contralateral SD	Ipsilateral Mean	Ipsilateral SD	p-value
Baseline	83.942	5.255	85.168	8.127	ns
Day 1	108.250	16.400	43.167	19.984	0.003577
Day 3	88.385	24.090	14.923	10.974	0.000315
Day 5	114.240	40.762	40.082	20.756	ns
Day 7	147.113	8.917	77.502	20.230	0.00056
Day 10	146.092	9.108	76.688	16.326	0.000282
Day 14	116.608	6.039	36.500	11.712	0.000033
Week 3	148.255	23.329	41.032	11.217	0.000226
Week 4	149.288	10.777	67.242	10.888	0.000013
Week 5	147.400	23.062	69.855	15.719	0.00298
Week 6	140.633	15.888	73.513	16.775	0.002191
Week 8	144.817	14.253	113.522	16.978	ns
Week 10	130.053	14.263	123.507	11.275	ns
Week 12	144.105	19.862	121.897	8.763	ns
Week 14	134.137	12.073	125.915	11.419	ns
Week 16	128.557	10.996	109.350	18.055	ns

S8. Contralateral to Ipsilateral Weight Ratio: Mobile Only.
Statistical analysis with one-way ANOVA with Geisser-Greenhouse correction and Dunnet's correction for multiple comparisons.

ANOVA table	F (DFn, DFd)	P value
Treatment (between columns)	F (2.734, 13.67) = 10.01	P=0.0011

Mean, Standard Deviation and p-value at each timepoint are given below:

Timepoint	Mean	SD	Comparison to Baseline: p-value
Day 1	0.216	0.175	0.3337
Day 3	0.056	0.047	0.0118
Day 5	0.137	0.056	0.0057
Day 7	0.276	0.077	0.0045
Day 10	0.266	0.113	0.0068
Day 14	0.135	0.031	<0.0001
Week 3	0.121	0.065	0.012
Week 4	0.216	0.074	0.0087
Week 5	0.254	0.107	0.0918
Week 6	0.337	0.107	0.1305
Week 8	0.592	0.170	0.0189
Week 10	0.825	0.167	0.009
Week 12	0.834	0.126	0.1881
Week 14	0.788	0.235	0.0847
Week 16	0.771	0.143	0.0082

S9. Contralateral to Ipsilateral Area Ratio: Mobile Only

Statistical analysis with one-way ANOVA with Geisser-Greenhouse correction and Dunnet's correction for multiple comparisons.

ANOVA table	F (DFn, DFd)	P value
Treatment (between columns)	F (2.883, 14.42) = 9.879	P=0.0009

Mean, Standard Deviation and p-value at each timepoint are given below:

Timepoint	Mean	SD	Comparison to Baseline: p-value
Day 1	2.033	0.615	0.0631
Day 3	2.495	0.790	0.0403
Day 5	1.775	0.471	0.0993
Day 7	1.683	0.279	0.0021
Day 10	1.555	0.403	0.0837
Day 14	2.175	0.553	0.027
Week 3	2.405	0.568	0.0186
Week 4	2.052	0.263	0.0009
Week 5	1.998	0.514	0.032
Week 6	1.757	0.242	0.0071
Week 8	1.228	0.172	0.4914
Week 10	1.097	0.222	0.9972
Week 12	1.232	0.243	0.4741
Week 14	1.203	0.299	0.4704
Week 16	1.035	0.143	>0.9999
